# Insulin therapy contributes to the increased risk of colorectal cancer in diabetes patients: a meta-analysis

**DOI:** 10.1186/1746-1596-8-180

**Published:** 2013-10-31

**Authors:** Lei Wang, Shuang Cai, Zan Teng, Xin Zhao, Xinyue Chen, Xiaojuan Bai

**Affiliations:** 1 Department of Gerontology and Geriatrics, the First Hospital of China Medical University, NO.155, North Nanjing Street, Heping District, Shenyang 110001, China; 2 Department of Pharmacy, the First Hospital of China Medical University, NO.155, North Nanjing Street, Heping District, Shenyang 110001, China; 3 Department of Medical Oncology, the First Hospital of China Medical University, NO.155, North Nanjing Street, Heping District, Shenyang 110001, China

## Abstract

**Background:**

Recent epidemiological studies suggest that treatment with insulin may promote cancer growth. The present systematic review and meta-analysis of published observational studies was conducted to assess the risk of cancer during treatment with insulin.

**Materials and methods:**

A compressive search was conducted through MEDLINE, PubMed, Web of Science, EMBASE, and Chinese Biomedical Literature databases (CBM). Pooled relative risks (RRs) and 95% confidence intervals (CIs) were calculated with a random-effects model.

**Results:**

A total of four studies with one case-controls study and three cohort studies comparing the insulin therapy and colorectal cancer susceptibility were identified. When all four studies were analyzed, the summary RRs were 1.61 (95% CI = 1.18–1.35) in a random-effects model for individuals with insulin therapy, compared with individuals without insulin therapy, which suggests a statistically significant association between insulin use and colorectal cancer.

**Conclusions:**

Our findings provides the evidence that insulin therapy may contribute to the risk of colorectal cancer.

**Virtual slides:**

The virtual slide(s) for this article can be found here: http://www.diagnosticpathology.diagnomx.eu/vs/9339731010859509

## Introduction

Colorectal cancer (CRC) is a leading cause of cancer-related morbidity and mortality in the Western world. Obesity, Western-style diet, and lack of physical activity are established risk factors for CRC [1]. Few epidemiologic studies have evaluated whether insulin treatment is associated with risk of CRC [2-4]. Two retrospective studies, with clinical data from the United Kingdom, reported roughly 2-fold higher odds of CRC among type 2 DM patients who used insulin [2,4]. There was a null association between insulin use and CRC risk in a similar retrospective study conducted with data from US pharmacies [3]. An effect of insulin on colon carcinogenesis is biologically plausible. In studies conducted with rats, insulin administration appears to promote colon cancer growth and to increase proliferation in colonic epithelial tissue [5,6]. Whether insulin treatment increases risk of CRC is an important question because almost all patients with type 2 DM will eventually require insulin treatment [7].

In line with these considerations, it has been hypothesized that insulin use might influence CRC development. In the past years, several eligible case–control or cohort studies were performed to identify the association of insulin use with CRC risk. However, the results remain inconclusive and inconsistent. To date, no meta-analysis has been conducted to investigate the association between insulin therapy and CRC susceptibility. Hence, a meta-analysis based on a total of four independent studies was performed, which may provide the evidence for association of insulin therapy with CRC susceptibility.

## Methods

### Search strategy

We conducted a comprehensive search using key words “DM”, “diabetes”, “CRC”, “colorectal neoplasm”, “colon cancer”, “colon neoplasm”, “rectal cancer”, “rectal neoplasm”, “insulin” and “insulin therapy” in the following key electronic biomedical databases MEDLINE, PubMed, Web of Science, EMBASE, and Chinese Biomedical Literature databases (CBM) without date and language restrictions, and all eligible studies were detected before September 2013. Their reference lists were hand-searched to find other relevant publications. Titles and available abstracts were scanned for relevance, identifying papers requiring further consideration. Of the studies with overlapping data published by the same investigators, only the most recent or complete study was included in this meta-analysis.

### Eligibility and exclusion criteria

To be included in the meta-analysis, a published study had to meet the following criteria: (1) only original articles of a quantitative assessment of the relationship of insulin therapy and risk of colorectal cancer, (2) cohort studies, (3) adult human population, and (4) insulin or one of insulin products as the main independent variable, (5) results expressed as relative risk (RR), (6) studies with a 95% CI for RR, or sufficient data to calculate these numbers. While for the exclusion criteria, we provided as follows: (1) case–control or case-only studies, family-based studies, case reports, editorials, and review articles (including meta-analyses); (2) controls with other types of tumors, and (3) studies that did not provide data that allowed calculation of standard errors for effect estimates and if the estimates were not adjusted for age and gender were excluded. Moreover, when there were multiple publications from the same population or cohort, only data from the most recent report were included. In studies with overlapping cases/controls, the higher quality score, or the study with more information on origin of cases/controls was included in the meta-analysis.

### Data extraction

Information was carefully extracted from all eligible publications independently by two authors according to the inclusion criteria listed above. Disagreement was resolved by discussion between the two authors. The following data were collected from each study: the first author’s last name, year of publication, and country of population studied, study design, number of exposed and unexposed subjects, follow-up period, age, gender, type of DM (type 2 or combined type 1 and type 2), risk estimates with their corresponding confidence intervals, and variables controlled for by matching or in the multivariable model, numbers of cases and controls with the insulin therapy and CRC, respectively. For each study, we extracted the risk estimates that reflected the greatest degree of control for potential confounders. We did not limit the number of patients to include a study in our meta-analysis.

### Statistical analysis

We used the crude ORs or RRs with their corresponding 95% CI as the metric of choice. Based on the individual ORs or ORs, the pooled OR was estimated. Summary RR estimates with their corresponding 95% CIs were derived by the method of DerSimonian and Laird [8] using both fixed and random effects models. The fixed effects model was used when there was no heterogeneity of the results of studies; otherwise, the random-effects model was used. To take into account the possibility of heterogeneity across the studies, a statistical test for heterogeneity was performed using the Q statistic. The heterogeneity was considered significantly when P was below 0.10. It was assessed using the *I*^*2*^ statistic, which takes values between 0% and 100% with higher values denoting greater degree of heterogeneity (*I*^*2*^ = 0–25%: no heterogeneity; *I*^*2*^ = 25–50%: moderate heterogeneity; *I*^*2*^ = 50–75%: large heterogeneity; *I*^*2*^ = 75–100%: extreme heterogeneity) [9]. To assess sources of heterogeneity, we conducted a meta-regression and subgroup analyses. Publication bias was assessed by visual inspection of funnel plot [10]. Formal statistical assessment of funnel plot asymmetry was done with Egger’s regression asymmetry test and adjusted rank correlation test [11]. In addition, Begg’s adjusted rank correlation test and the trim-and-fill method were used [11,12]. Sensitivity analysis was used to explore the extent to which inferences might depend on a particular study or group of studies. Statistical analyses were carried out with Stata, version 12.0 (Stata Corp, College Station, TX, USA) and Review Manager (version 5.0). P < 0.05 were considered statistically significant. All statistical tests were two-sided.

## Results

### Study characteristics and meta-analyses

Through literature search and selection, a total of four studies with one case-controls study and three cohort studies [2,4,13,14] comparing the insulin therapy and CRC susceptibility were identified. Chung et al. performed a case–control study, including 100 CRC patients and 225 controls, all the subjects are of type 2 diabetes [13]. Cambell et al. listed 2,809 patients, exposed and comparing group were 11,335 and 143,660 subjects, respectively. All these subjects are also of type 2 diabetes [14]. Currie et all listed 292 CRC patients, exposed and comparing group were 10,067 and 52,742 subjects, respectively. All these subjects are also of type 1 and 2 diabetes [4]. Yang et al. listed 125 patients, exposed and comparing group were 3,160 and 21,758 subjects, respectively. All these subjects are also of type 2 diabetes [2]. Of these four studies, one was from Korea [13], while one were from UK [2,4], and the other from USA [14]. When all four studies were analyzed, the summary RRs were 1.61 (95% CI = 1.18–1.35) in a random-effects model for individuals with insulin therapy, compared with individuals without insulin therapy, which suggests a statistically significant association between insulin use and colorectal cancer.

### Sensitivity analysis

In order to compare the difference and evaluate the sensitivity of the meta-analysis, we conducted one-way sensitivity analysis to evaluate the stability of the meta-analysis. The statistical significance of the results was not altered when any single study was omitted, confirming the stability of the results (data was not shown). Hence, results of the sensitivity analysis suggest that the data in this meta-analysis are relatively stable and credible after exclusion small size of studies.

### Publication bias

For publication bias assessing, Begg’s and Egger’s test were employed. Begg’s funnel plot was performed to assess the publication bias of the literature. The shapes of the funnel plots did not reveal significant evidence of obvious asymmetry. Furthermore, Egger’s test was used to provide statistical evidence for funnel plot symmetry. The results still did not suggest any evidence of publication bias.

## Discussion

In this present work, one case–control study [13] and three cohort studies [2,4,14] showed that insulin therapy was associated with risk of CRC. To the best of our knowledge, it is the first systematic review that has investigated the association of insulin use and CRC susceptibility. Chung et al. [13] found patients who received chronic insulin therapy had three times the risk of CRC compared with patients who received no insulin (OR = 3; 95% CI = 1.1–8.9). Yang et al. [2] found chronic insulin therapy significantly increases the risk of CRC among type 2 DM patients (HR = 2.1; 95% CI = 1.2–3.4). Currie et al. [4] found insulin therapy increased the risk of CRC compared with metformin therapy (HR = 1.69, 95% CI = 1.23–2.33). Whereas, Campbell et al. [14] observed insulin use was not associated with a substantially increased risk of CRC (RR = 1.05; 95% CI = 0.82–1.36). In our present meta-analysis, the summary RRs were 1.61 (95% CI = 1.18–1.35) in a random-effects model for individuals with insulin therapy, compared with individuals without insulin therapy.

Similar to other systematic reviews and meta-analysis, our study also has some limitations: (1) small sample size is limitation in our work, (2) publication bias may be present, although we have used our best efforts to identify relevant reports/articles, (3) most of the studies did not distinguish between types 1 and 2 diabetes, (4) diabetes is an under-diagnosed disease, and some degree of misclassification of exposure to diabetes is likely to have occurred, and (5) meta-analysis is just a statistical test that is subject to many methodological restrictions and is not able to control for other relevant factors.

Some limitations listed above, there are also some advantages should be emphasized. Our results may have important clinical and public health implications. DM is a serious and growing health problem worldwide. CRC, one of the most common malignancies of the gastrointestinal tract, is a significant health problem. Meta-analysis provided a popular method for combining world literatures across studies to resolve the statistical power and discrepancy problem in associate studies [15-18]. The growing worldwide frequency of diabetes will probably increase as a result of the insulin or insulin related products therapy, and thus this disease may contribute to the development of additional cases of CRC. In order to acquire a more rational approach to CRC prevention and treatment in diabetic patients, more evidence and mechanisms needs to be properly obtained.

In conclusion, our analysis suggests that insulin therapy will significantly increase the risk of colorectal cancer. More well-designed with larger sample studies will be needed to verify our results.

## Competing interests

The authors declare that they have no competing interests.

## Authors’ contributions

LW, SC, ZT, XZ, XC, and XB carried out the meta-analysis study, drafted the manuscript and involved in revising the manuscript critically for important intellectual content. LW, SC, ZT, and XB participated in the design of the study and revised the manuscript. LW, XZ, XC, and XB carried out the meta-analysis study and drafted the manuscript. LW, SC, ZT, XZ, XC, and XB participated in the design of the study, drafted the manuscript and revised the manuscript. All authors read and approved the final manuscript.
